# Discrete wavelet transform de-noising in eukaryotic gene splicing

**DOI:** 10.1186/1471-2105-11-S1-S50

**Published:** 2010-01-18

**Authors:** Tina P George, Tessamma Thomas

**Affiliations:** 1 Department of Electronics and Instrumentation, College of Engineering, Kidangoor, Kottayam, Kerala, India; 2 Department of Electronics, Cochin University of Science And Technology, Kerala, India

## Abstract

**Background:**

This paper compares the most common digital signal processing methods of exon prediction in eukaryotes, and also proposes a technique for noise suppression in exon prediction. The specimen used here which has relevance in medical research, has been taken from the public genomic database - GenBank.

**Methods:**

Here exon prediction has been done using the digital signal processing methods viz. binary method, EIIP (electron-ion interaction psuedopotential) method and filter methods. Under filter method two filter designs, and two approaches using these two designs have been tried. The discrete wavelet transform has been used for de-noising of the exon plots.

**Results:**

Results of exon prediction based on the methods mentioned above, which give values closest to the ones found in the NCBI database are given here. The exon plot de-noised using discrete wavelet transform is also given.

**Conclusion:**

Alterations to the proven methods as done by the authors, improves performance of exon prediction algorithms. Also it has been proven that the discrete wavelet transform is an effective tool for de-noising which can be used with exon prediction algorithms.

## Background

**Figure 1:**
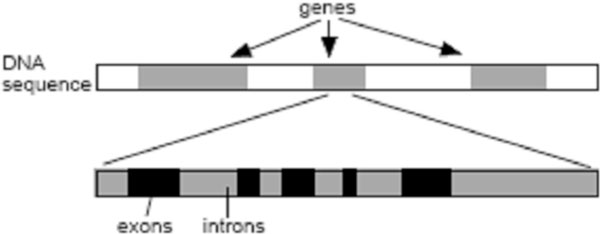
**Introns and exons in a DNA sequence**. A DNA sequence has genes as well as inter-geneic spaces(shown in white) in it. The genes in turn are made of introns and exons.

Genes in eukaryotic cells have two sub-regions, exons and introns [1], depicted in Figure 1. A preliminary step in the analysis of genomic data, known as DNA-splicing or exon prediction, determines the locations of the exons. The four bases of each strand of the DNA double-helix - Adenine, Thymine, Guanine, and Cytosine are represented distinctly in a genomic sequence with the letters A, T, C, and G to [1]. Protein-coding regions in a DNA sequence-exons (Figure 1) exhibit a period-3 property [1] because of the codon structure involved in the translation of base sequences into amino acids [2,3]. The period-3 property is in general regarded as a good preliminary indicator of exon locations, although there are certain exceptions [2]. Digital Signal Processing (DSP) techniques which exploit this period 3 property for exon prediction make use of DSP tools like the Discrete Fourier transform (DFT) [4] or bandpass digital filters [5]. Trevor W. Fox and Alex Carreira [6] have proposed a method of reduced computation to map out exons in a genomic sequence, suppressing noise to a greater degree. But the drawback of all these methods is the continued presence of inter-exon noise. We have used the Discrete Wavelet Transform (DWT) to achieve greater noise suppression [7]. To design any exon prediction algorithm, first step is to convert the sequences of letters from the four-character alphabet into binary sequences conducible to digital signal processing. The numerical sequence resulting from a character string of length N can be written as(1)

*u*_*A *_[*n*], *u*_*T *_[*n*], *u*_*C *_[*n*], and *u*_*G*_[*n*] are the *binary indicator sequences*, which take the value of either 1 or 0 at location *n*, depending on whether the corresponding character exists or not, respectively, at *n*. Here we have taken values of a, t, c, g as 1. The string ACCTG has *N *= 5 and is called the *length *of the sequence. Also,(2)

## Methods

Many digital signal processing methods have been tried for genomic data analysis with proven results [1,4-6,9,10], are but a few examples of such published work.

### Exon prediction using the DFT - The binary method

This method [4] uses the binary indicator sequences obtained as described above, the DSP tool used being the DFT. As per the classical definition [11], DFT of a sequence *x*[*n*], of length *N*, is itself another sequence *X*[*k*], of the same length *N*.(3)

The sequence *X*[*k*] provides a measure of the frequency content at "frequency" *k*, which corresponds to an underlying period of *N/k *samples. Using the above definition the *U*_*A*_[*k*] , *U*_*T*_[*k*] , *U*_*C*_[*k*], and *U*_*G*_[*k*] are the DFTs of the binary indicator sequences *u *_*A*_[*n*], *u*_*T*_[*n*], *u*_*C*_[*n*], and *u*_*G*_[*n*], respectively and then it follows that:(4)

As already mentioned here, a = t = c = g = 1.(5)

has been used as a measure of the total power spectral content of the DNA character string, at "frequency" *k*. But we've found [8] that(6)

gives better results than the one given in equation 5. The period-3 property of a DNA sequence implies that the DFT coefficients corresponding to *k *= *N*/3 is large. Thus if we take *N *to be a multiple of 3 and plot S[*k*] then we should see a peak at the sample value *k *= *N*/3. Instead of evaluating the DFT of a full-length sequence, DFTs of several of its subsequences, (STFT) was computed for better time domain resolution by sliding the window by one entry in the sequence.

### Exon prediction using the DFT - The EIIP method

In this method, described in [11], letters of the DNA sequence A, T, C, G are replaced with the electron ion interaction pseudo-potentials(EIIP) of nucleotides. If we substitute the EIIP values in x[n], we get a numerical sequence the 'EIIP indicator sequence', x_e_[n] which represents the distribution of the free electrons' energies along the DNA sequence, for A, T, C, G the values are 0.126, 0.1335, 0.134, 0.0806 respectively. Next, DFT is evaluated and the corresponding value of the power spectrum is(7)

When Se[k] is plotted against k, it reveals a peak at N/3 for a coding region and no such peak is observable for a noncoding region. Rectangular windows were used in this work, for evaluating the STFT by breaking up the long sequence into subsequences.

### Exon prediction using digital filters

Digital filtering methods used for identification of exons make use of the period-3 behaviour [5] coding regions. The output of an antinotch filter, with a sharp gain at the frequency 2*π*/3 provides this information as a function of base location. This filter [5] has an impulse response *w(n) *given by,(8)

**Figure 2:**
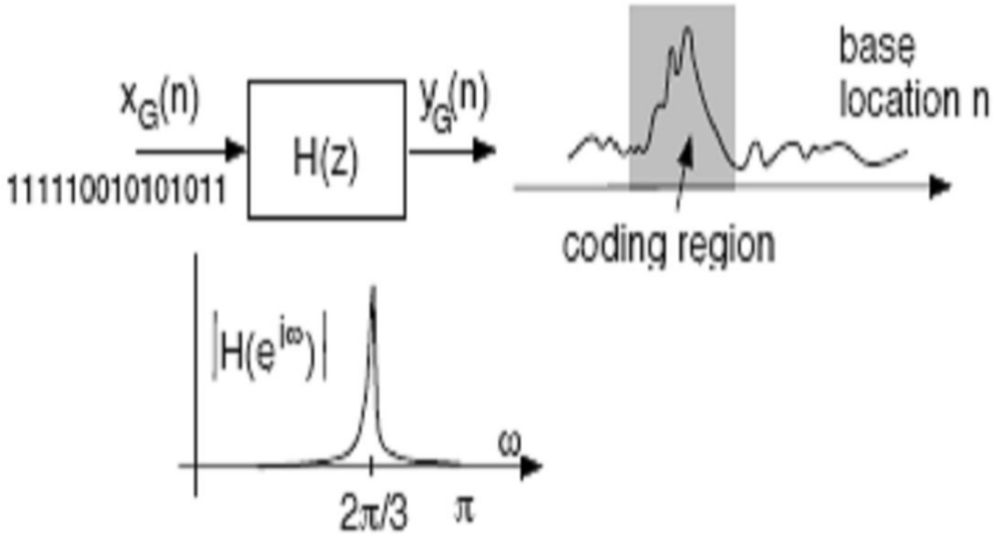
**Expected output of anti-notch filter *x*_*n*_(*G*) - indicator sequence, *H*(*z*)-anti-notch filter with pass band centred at 2*π*/3, *y*_*n*_(*G*) - output of the filter**.

Let *H*(*z*) be a narrow band bandpass or anti-notch digital filter with a sharp passband cantered at *ω*_0 _= 2*π*/3. With the indicator sequence *x*_*G*_(*n*) taken as input, let *y*_*G*_(*n*) denote its output. In the coding regions, the sequence *x*_*G*_(*n*) is expected to have a period-3 component, which means that it has large energy in the filter passband. So the output *y*_*G *_(*n*) should be comparatively large in the coding regions as demonstrated in Figure 2. With similar notation for the other bases, define(9)

A plot of this function is a preliminary indicator of coding regions. Filter 2 designed by the authors [6] gives better result than this anti-notch filter-Filter 1, called so in this paper.

#### Filter 1

The design [5] starts by considering second order all-pass filter, *A(z)*.(10)

Now consider a filter bank with two filters *G*(*z*) and *H*(*z*) defined as,(11)

Then *G*(*z*) has the form(12)

*G*(*z*) is a notch filter with a zero at the frequency *w*_0_.(13)(14)

**Figure 3:**
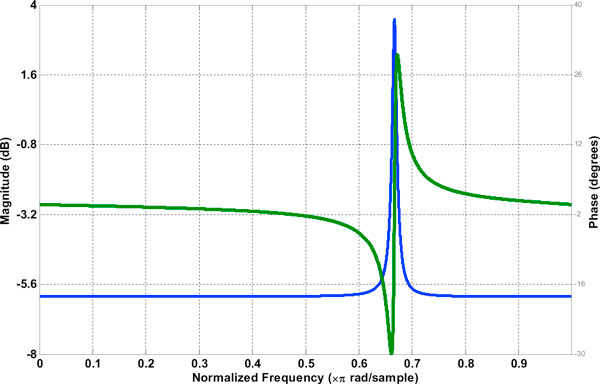
**Magnitude and phase responses of Filter 1**. The magnitude response is shown in blue and phase response in green.

H(z) is the required anti-notch filter with magnitude and phase responses as in Figure 3.

#### Filter 2

**Figure 4:**
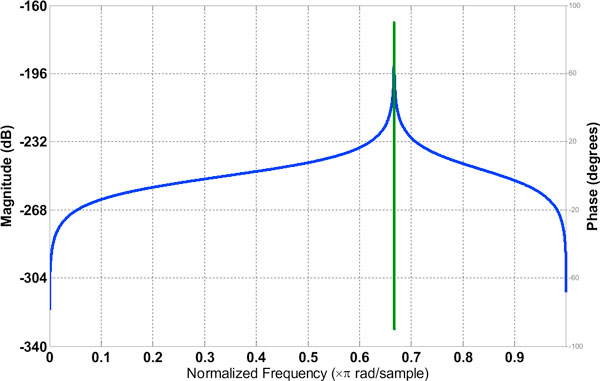
**Magnitude and phase responses of Filter 2**. The magnitude response is shown in blue and phase response in green.

Filter 2, in this paper is an IIR single peaking filter with the peak frequency at 2*π*/3. This was designed using the built-in utility of MATLAB [6]. Its magnitude and phase response is shown in Figure 4.

#### Reduced computation technique in filter method

The number of digital filter operations can be reduced from four to one [6] by creating a new signal that encapsulates the entire DNA sequence *u*_A+C+T+G_(*n*) = *au*_A_(*n*) + *cu*_C_(*n*) + *tu*_T_(*n*) + *gu*_G_(*n*) where *a*, *c*, *t*, and *g *are real-valued parameters.. A long DNA sequence can be approximated using a two-symbol representation, where one symbol is either A or T and the other symbol is either C or G as they are complimentary to each other. Also capitalizes on the strong periodicity exhibited by the G sequence. In this case, the signal becomes(15)

### DWT to improve gene splicing techniques

The above methods of gene splicing, though give results, better reduction in noise and accuracy of prediction is desired. The statistically optimal null filter to improve prediction of exons has been suggested by Kakumani et. al. [10]. Here we've tried to improve the accuracy of a gene splicing algorithm using the Discrete Wavelet Transform (DWT). In DWT [12], the signal is passed through a series of high and low pass filters to analyze the respective frequencies followed by a scaling. The scale is changed by upsampling and downsampling (subsampling) operations. Subsampling reduces the sampling rate, or removes some of the samples of the signal. Upsampling increases the sampling rate of a signal by adds new samples. Filtering involved is explained as follows. If a signal has a maximum of 1000 Hz component, then half band low-pass filtering removes all the frequencies above 500 Hz. However it is to be recalled that with discrete signals, frequency *ω *is expressed in terms of radians. Accordingly, the sampling frequency of the signal is equal to 2*F*_*m*_, Hz, in the analog domain and 2*π *radians in terms of discrete radial frequency. Therefore, the highest frequency component in a discrete signal will be *π *radians. Hz is not appropriate for discrete signals, but used for clarity of the idea.

**Figure 5:**
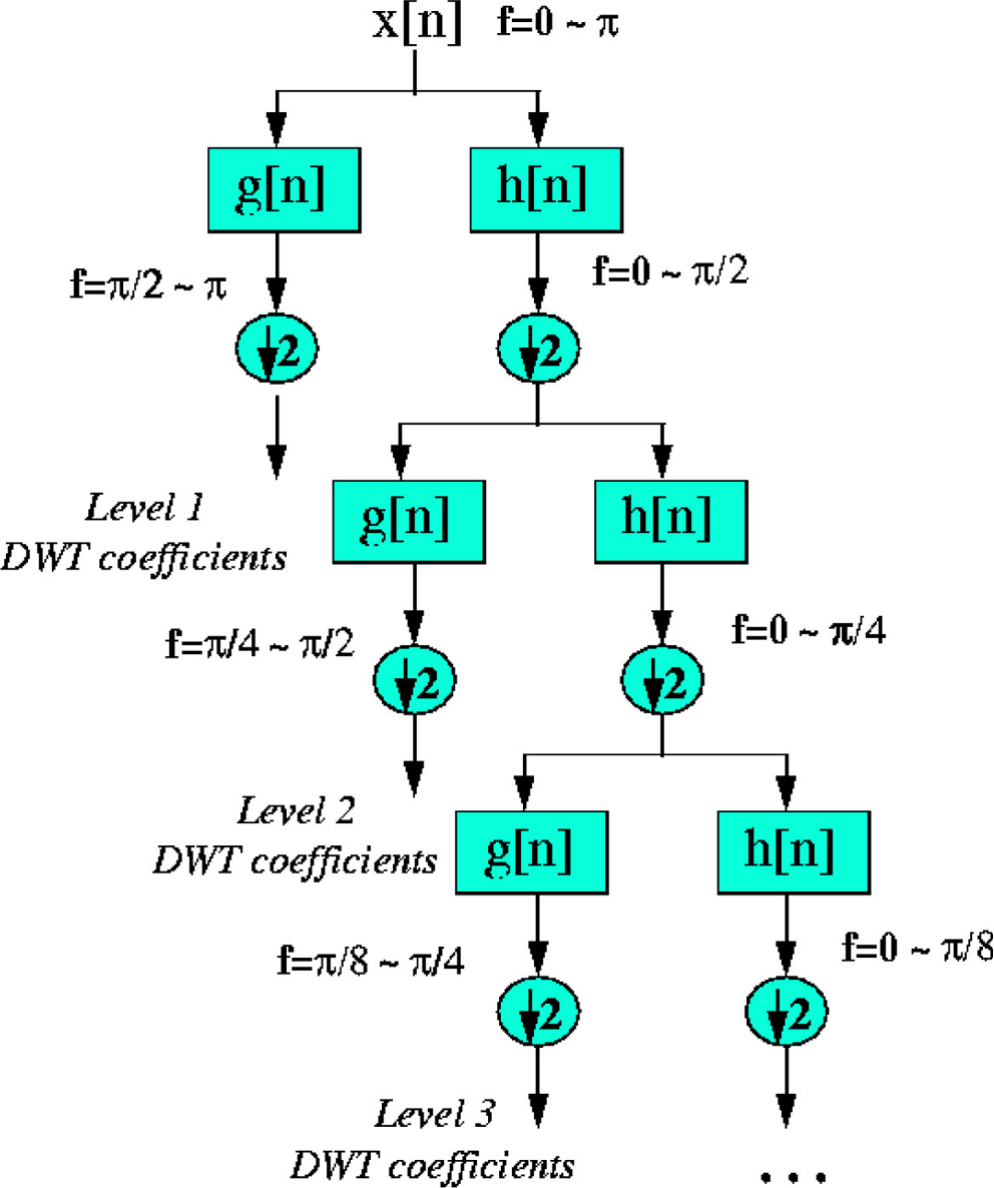
**DWT decomposition**. Schematic of DWT decomposition at 3 levels, h[n] - the low pass half band filter, g[n] - the high pass half band filter(notations are in discrete time domain).

Decomposition of the signal into different frequency bands is obtained by successive high pass and low pass filtering of the time domain signal. The original signal x[n] is first passed through a halfband, highpass filter g[n] and a lowpass filter h[n]. After the filtering, half of the samples can be eliminated ie. subsampled by 2, by discarding every other sample. This constitutes one level of decomposition, mathematically expressed as:(17)(18)

h[n] and g[n] are the sample sequences or impulse responses and y_high_[k] and y_low_[k] are the outputs of the highpass and lowpass filters, respectively, after subsampling by 2. This decomposition halves the time resolution since only half the number of samples now characterizes the entire signal. However, this operation doubles the frequency resolution, since the frequency band of the signal now spans only half the previous frequency band. The above procedure, known as subband coding, can be repeated for further decomposition. At every level, the filtering and subsampling will result in half the number of samples (and hence half the time resolution) and half the frequency band spanned (and hence double the frequency resolution). Figure 5 illustrates this procedure.

The bandwidth of the signal at every level is marked on the figure as "f". The DWT of the original signal is obtained by concatenating all coefficients starting from the last level of decomposition (remaining two samples, in this case) and will have the same number of coefficients as the original signal. The difference of this from the Fourier transform is that the time localization of these frequencies will not be lost, a key advantage. Good time resolution is obtained at high frequencies, and good frequency resolution at low frequencies. All algorithms mentioned in this work were implemented using MATLAB.

## Results

**Figure 6:**
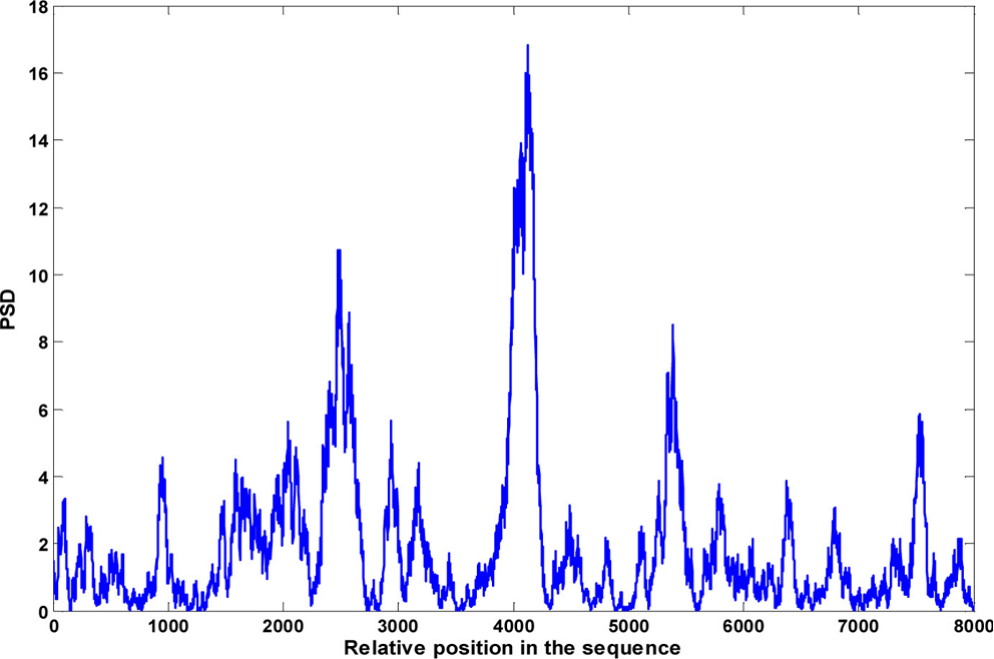
**Exon plot-1**. Result of Binary method (using the DFT), Window: 240.

**Figure 7:**
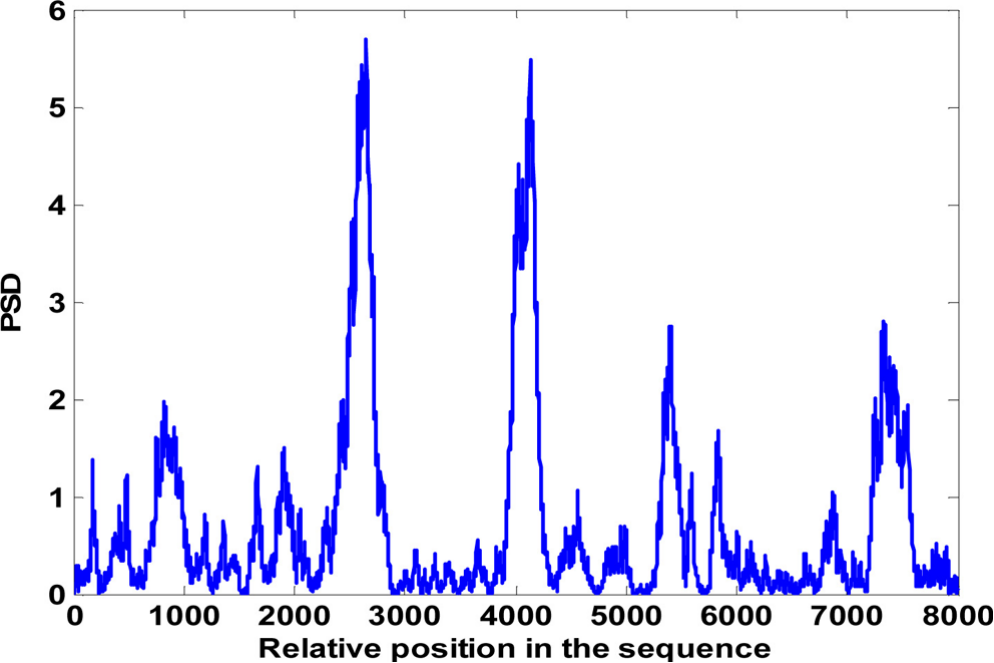
**Exon plot 2**. Result of EIIP method (using the DFT), Window: 240.

**Figure 8:**
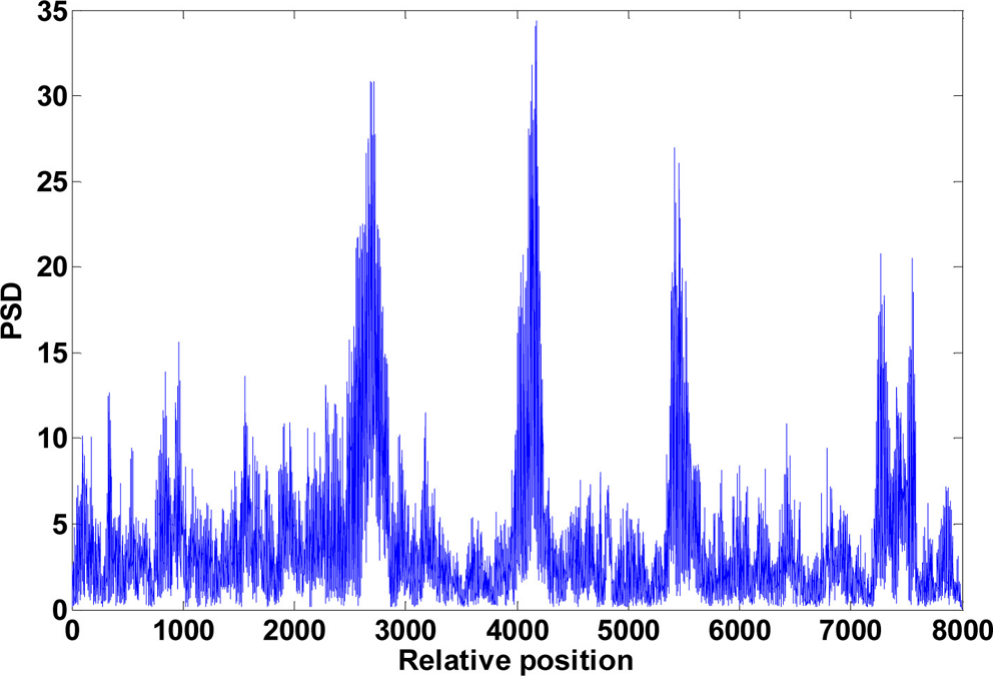
**Exon plot 3**. Result of Filter method using Filter1.

**Figure 9:**
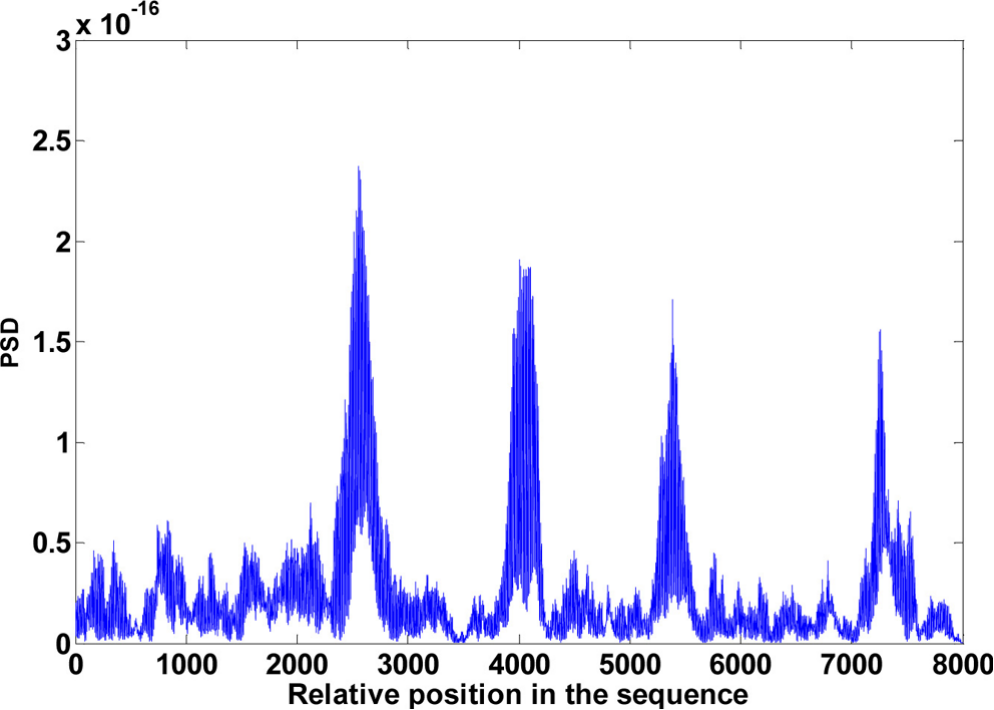
**Exon plot 4**. Result of Filter method, using Filter2, designed by the authors detailed in [8].

**Figure 10:**
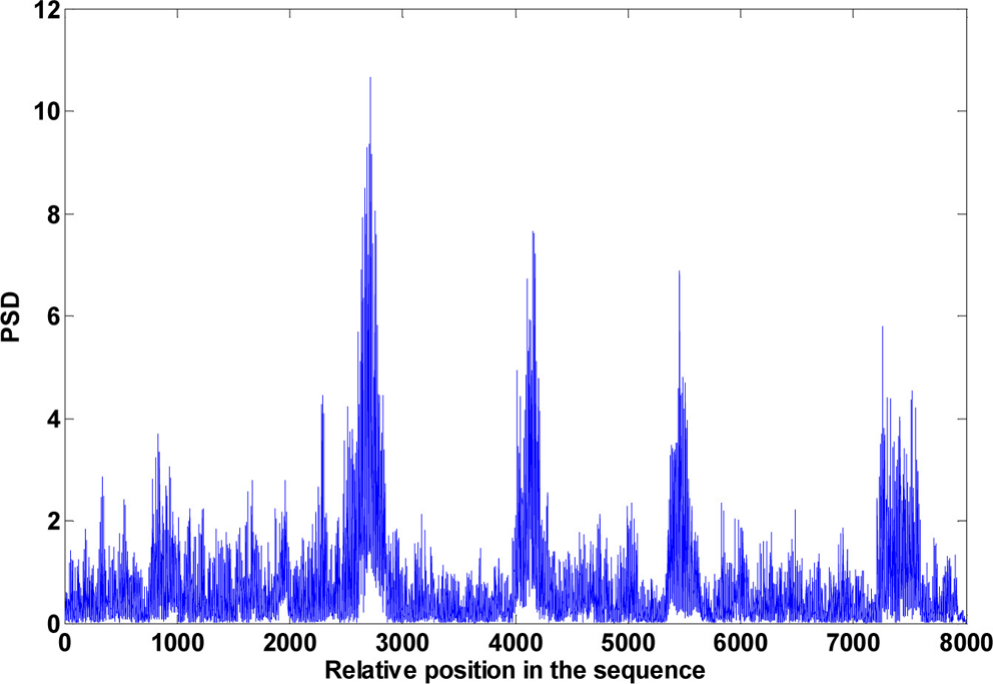
**Exon plot 5**. Result of the reduced computation method [6] using Filter1

**Figure 11:**
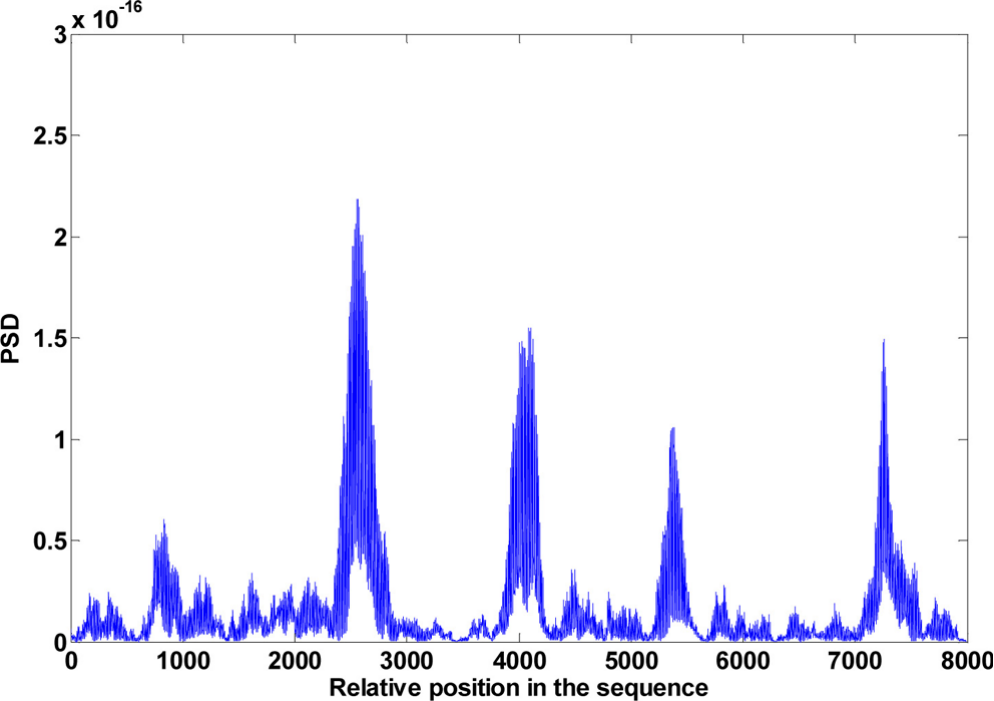
**Exon plot 6**. Result of the reduced computation method [6] using Filter2

**Figure 12:**
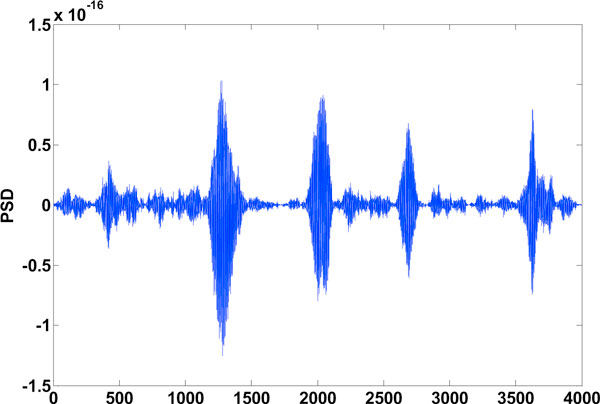
**High frequency components of level 1 DWT decomposition**. The high frequency components in the spectrum after level 1 DWT decomposition.

**Figure 13:**
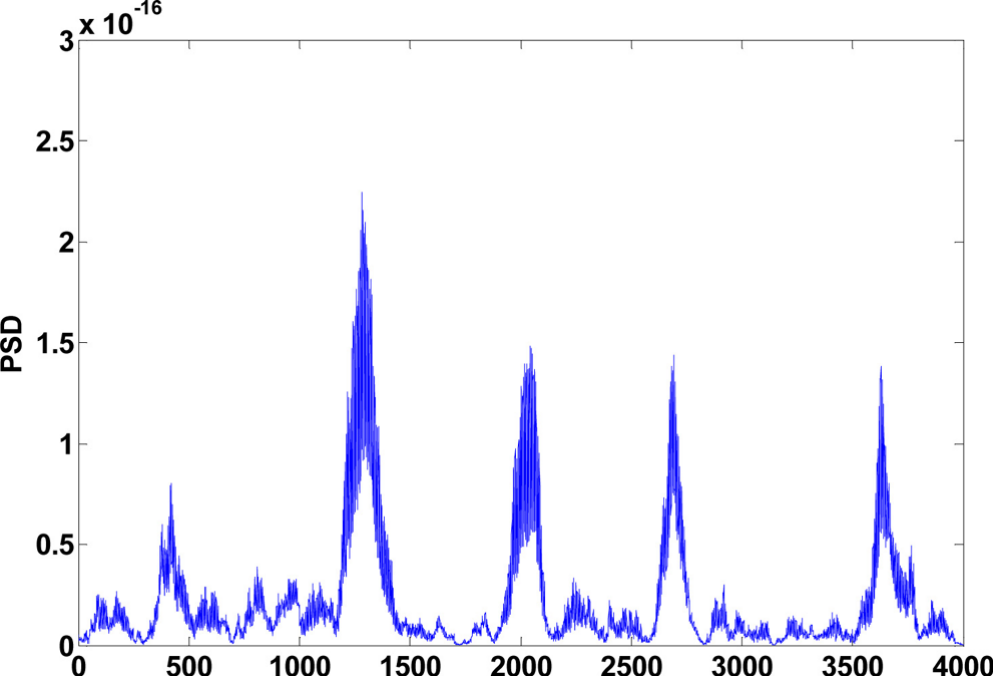
**Low frequency components of 1 level DWT decomposition**. The low frequency components in the spectrum after level 1 DWT decomposition

**Figure 14:**
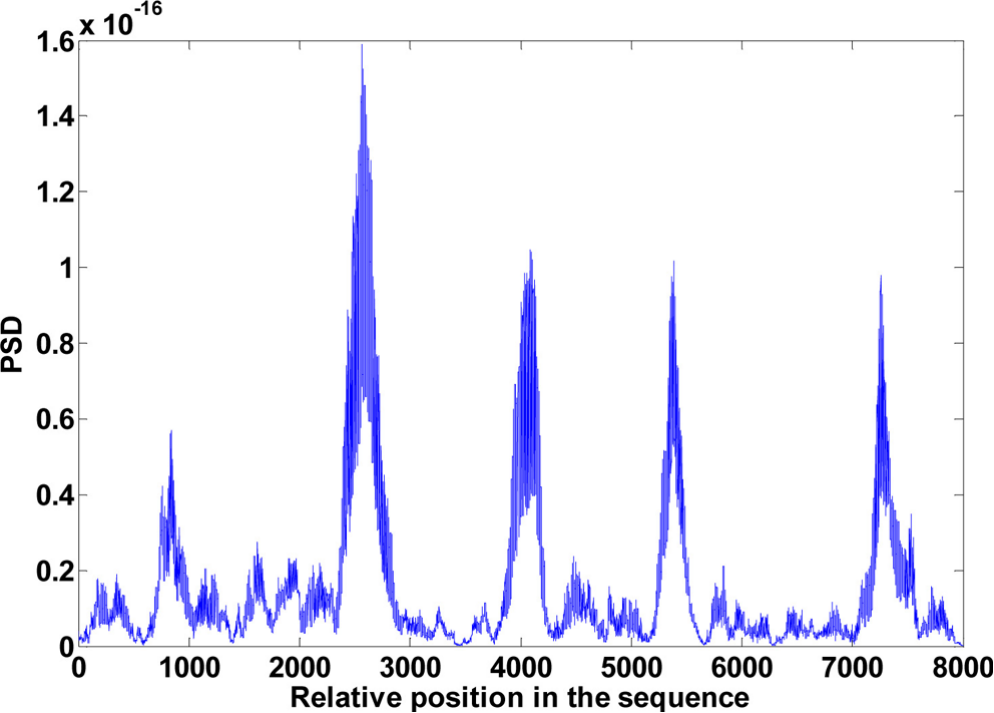
**Exon plot after DWT de-noising**. The exon plot with pronounced peaks after DWT de-noising.

Figures 6, 7, 8, 9, 10, 11, 12, 13, 14 are the results of the algorithms described in this work applied on exons in nucleotide sequence of the gene F56F11.5 of C elegans [GenBank: AF099922]. The authors have tried the DSP methods on genomic sequences of four different specimen - C elegans, Mus musculus, Sus scrofa and Homo sapien, but only the results obtained with organism C elegans has been included here due to lack of space. *Caenorhabditis elegans *is a free living nematode (roundworm), about 1 mm in length, which lives in temperate soil environments. The bases are 1...42799 long and 8000 nucleotides from location 7021 have been considered which according to the NCBI data base has five exons.

### Binary method and EIIP method

Results obtained are the exon plots shown in Figures 6 and 7 respectively. Of the gene splicing algorithms mentioned here, the ones which make use of the DFT are the Binary method and the EIIP method. C elegans gives best result for a window length of 240. The boundary of exons is more well defined with this window. A window size of 351 though reduces inter-exon noise, the exon boundaries tend to shift, its not shown here.

### Filter method

The results obtained with digital filtering is shown in Figures 8 to 11. The filter designed by the authors named Filter 2 here, gives better results than Filter 1[5]. The noise suppression technique with reduced computation [6] reduces inter-exon noise to a great. Exon plot 5 shows that even the noise suppression technique when used with the filter 1 fails to give the desired results, but very effective when used in conjunction with the filter 2 as seen from exon plot 6 (Figure 11). But even then, the first exon located between 7947 and 8059, with relative position in the plot, 926 - 1079 cannot be distinguished from the surrounding inter-exon noise. The noise peaks as seen in the exon plot 5 (Figure 10, reduced computation technique with filter 2) are stronger than the half power values of the exon peaks. It's evident that these methods need improvement. Hence DWT de-noising has been tried. The best results as obtained from reduced computation technique with IIR anti-notch filtering using filter 2 was used for further treatment with DWT. All the results are shown in tabular form in table 1 against standard exon lengths give in the NCBI database.

**Table 1:** Tabulation of results. Exon locations of [GenBank:AF099922] as given in the NCBI database, and those obtained using the various DSP methods discussed here.

Exon locations obtained for C elegans						
**Binary method**	**EIIP method**	**Filter 1**	**Filter 2**	**Reduced computation with Filter 1**	**Reduced computation with Filter 2**	**NCBI ranges**

7921-8021(100)	7821-8021(200)	7921-8021(100)	7821-8021(200)	7821-8021(200)	7841-8021(180)	7947-8059(112)
9521-9821(300)	9521-9821(300)	9521-9821(300)	9521-9851(330)	9521-9871(350)	9521-9851(330)	9548-9879(331)
11021-11221(200)	11021-11221(200)	11021-11221(200)	10921-11221(300)	11021-11271(250)	10921-11221(300)	11134-11397(263)
12321-12521(200)	12421-12621(200)	12421-12621(200)	12321-12541(220)	12321-12521(200)	12321-12541(220)	12485-12664(179)
14281-14621(340)	14221-14621(400)	14221-14621(400)	14221-14621(400)	14221-14621(400)	14221-14621(400)	14275-14625(350)

### DWT to improve gene splicing techniques

Figure 12 mentioned in the results section shows the detail coefficients and Figure 13 shows the approximation coefficients of Haar decomposition respectively. The final exon plot obtained after DWT treatment are given in Figure 14. Notice that the in exon plot 6, Figure 11 power levels corresponding to the first exon which had half power values almost equal to the noise levels (exon plot 6) has been accentuated such that there is no mistaking between exon region and intron region. As the signals desired corresponding to exon peaks are in the lower region of the spectrum spanning the 0 - *π*/2 range, against a discrete frequency interval of - *π *to *π*, a single level decomposition and reconstruction was sufficient here. As already mentioned, the region of the genomic sequence of C elegans has 8000 nucleotides from 7021 to 15021. The exon plots in figures 6 to figure 14 show 8000 nucleotide locations with the exons depicted as spectral peaks. The exon boundaries obtained after de-noising with DWT are the same as those obtained with the reduced computation technique using Filter2, as the the exon plot obtained with the method was used for subsequent wavelet decomposition and re-construction. Hence the exon boundaries are not tabulated separately for the de-noised result.

## Discussion

DFT is a conventional frequency analysis tool. Instead of evaluating the DFT of a full-length sequence, the DFTs of several of its subsequences, ie. the STFT was computed for better time domain resolution by sliding the window by one entry in the sequence. It is a known fact that using the STFT increases resolution in time domain. For the first two methods, most of the literature asserts 351 to be the window size, especially for C elegans. But the authors have found that the window size varies with the method adopted and the DNA sequence analyzed. With the DFT used for frequency analysis, the window found to yield better result was 240. The better result obtained with the single peaking IIR filter over the one described in [5] can be attributed to the higher attenuation seen in the stop band of the filter. The use of such a filter has given lesser noise without using the subsequent filter bank mentioned in [5]. DWT is a far more popular and potential signal processing tool today. However it has been used only for noise suppression here. Review of literature did not reveal a formal, randomized comparison of each of engineering methods mentioned here with other non-engineering approaches, hence such a comparison is not presented.

## Conclusion

In this paper the authors have shown that appropriate alterations to the classical methods of exon prediction yields better results. For AF099922 C elegans, the window size for the binary and EIIP methods has been found to be 240, whereas for the digital filter method it is 450, as against 351 mentioned in most of the literature. The window size thus should be selected depending on the method of analysis and also on the sequence analyzed. The filter1 as it is called in this paper is the common filter found in literature [3,5]; filter 2 has been designed by the authors. It's clear that this design is much better performance-wise as evident from the results. We have proposed the DWT to de-noise exon prediction, and it has been proved that it is the right tool for de-noising to be used with exon prediction algorithms.

## Competing interests

The authors declare that they have no competing interests.

## Authors' contributions

TPG carried out the work and drafted the manuscript under the close guidance of TT who conceived of the study, and participated in its design and coordination. Both authors have read and approved the final manuscript.
